# Molecular Characterization of *Giardia lamblia*: First Report of Assemblage B in Human Isolates from Rio de Janeiro (Brazil)

**DOI:** 10.1371/journal.pone.0160762

**Published:** 2016-08-12

**Authors:** Clarissa Perez Faria, Graziela Maria Zanini, Gisele Silva Dias, Sidnei da Silva, Maria do Céu Sousa

**Affiliations:** 1 CNC - Center for Neurosciences and Cell Biology, University of Coimbra, Coimbra, Portugal; 2 Faculty of Pharmacy, University of Coimbra, Coimbra, Portugal; 3 Laboratory of Parasitology, Evandro Chagas National Institute of Infectious Diseases, Oswaldo Cruz Foundation, Rio de Janeiro, Brazil; NIH, UNITED STATES

## Abstract

**Background:**

Despite the high prevalence of giardiasis, the genetic characterization of *Giardia lamblia* has been poorly documented in Brazil and molecular epidemiology research has only been conducted in the last few years. The aim of this study was to determine the prevalence of different *G*. *lamblia* assemblages and detect mixed infections among patients with giardiasis.

**Methods and Principal Findings:**

The cross-section survey was conducted among patients attending the FIOCRUZ in Rio de Janeiro. In order to discriminate the genetic assemblages/sub-assemblages, *G*. *lamblia* isolates were characterized by PCR-RFLP and qPCR using four loci genes (*bg*, *gdh*, *tpi* and *orf*C4). Of the 65 positive samples, 41 (63.1%) were successfully amplified by nested-PCR of *bg* and *gdh* genes. Among them, 16 were typed as sub-assemblage AII, 7 as BIII, 4 as BIV and 8 as a mixture of BIII and BIV. After the analysis by qPCR assay, a total of 55 (84.6%) samples were amplified using at least one locus: *bg* gene was amplified in 38 (58.5%) samples, *gdh* in 41 (63.1%), *tpi* in 39 (60%), and *orf*C4 in 39 (60%). Multilocus genotyping results showed that 29 (52.7%) samples belonged to Assemblage A and 26 (47.3%) samples belonged to Assemblage B. In 2011 and 2012, 20 (74.1%) samples belonged to Assemblage A and 7 (25.9%) belonged to Assemblage B. In subsequent years (2013–2015) there was a predominance of Assemblage B, 19 (67.9%) *versus* 9 (32.1%) Assemblage A.

**Conclusions:**

This is the first time that Assemblage B of *G*. *lamblia* was reported in human clinical samples from Rio de Janeiro (Brazil) and is the first report about genetic characterization using four genes. The qPCR assemblage-specific showed no mixed infections by Assemblages A and B. A switch in genetic profile over the years was observed, firstly predominance of Assemblage A and lastly of Assemblage B.

## Introduction

*Giardia lamblia* (syn. *G*. *intestinalis*, *G*. *duodenalis*) is one of the most frequent human intestinal protozoa reported worldwide, with estimated prevalence rates of 20–30% in developing countries and 2–5% in industrialized countries [1]. Giardiasis has been included in the Neglected Diseases Initiative of the World Health Organization (WHO) since 2004 due its impact on health [2].

*G*. *lamblia* is considered a species complex, whose members show little variation in their morphology but presents a remarkable genetic variability. This species is divided into eight distinct genetic assemblages (A-H), but only Assemblages A and B are known to infect humans. The remaining assemblages are likely to be host specific, as Assemblages C and D predominantly occur in dogs and other canids, Assemblage E in hoofed livestock, Assemblage F in cats, Assemblage G in rats and Assemblage H in marine mammals [3, 4]. Assemblage A was subdivided into three sub-assemblages: AI is preferentially found in animals; AII is commonly found in humans, although it was reported in a few studies in animals; and sub-assemblage AIII is exclusively found in animals. The host distribution of Assemblage B, which was subdivided into two sub-assemblages BIII and BIV, is predominantly human and much less common in animals [5, 6].

Mixtures of assemblages in individual isolates have often been observed, and the frequency of mixed infections may be underestimated by the use of a single marker [5]. The application of assemblage-specific primers coupled with the use of more than one molecular marker has been employed to assess, more accurately, the occurrence of mixed infections in clinical samples and to improve the detection of assemblages [1, 7, 8]. Until now the molecular analysis of *G*. *lamblia* samples at the β-giardin (*bg*), glutamate dehydrogenase (*gdh*) and triose phosphate isomerase (*tpi*) genes confirmed the high genetic variability within Assemblages A and B [9].

Assemblages A and B have been considered genetic variants of the same species. However, the latest studies suggest that the genomic differences between Assemblages A and B are sufficient to classify them into two different species [10, 11]. Certainly, the understanding of the epidemiology of giardiasis is committed by the uncertainty of taxonomy.

In Brazil the prevalence of giardiasis varies dramatically between different regions of the country mainly due to its enormous expanse of territory [12, 13]. Despite the high prevalence of the infection, the genetic characterization of the parasite has been poorly documented and molecular epidemiology research has been conducted in the last few years [12, 14–19]. Most of the epidemiological studies detected *G*. *lamblia* on the basis of microscopic examination [13, 20–22]. So far only one study was performed with Rio de Janeiro samples [23], consequently the *G*. *lamblia* assemblages in this city are poorly known. The objective of this study was to determine the prevalence of different *G*. *lamblia* assemblages and sub-assemblages among patients with giardiasis attending a referral hospital in Rio de Janeiro, therefore providing additional information on the molecular epidemiology of this parasite in the country. The study also aimed to determine the occurrence of mixed infections using primers targeting *gdh*, *tpi* and *orf*C4 genes specific for Assemblages A and B.

## Methods

### Stool samples collection and laboratory methods

The cross-section survey was carried out from January 2011 to February 2015 in Evandro Chagas National Institute of Infectious Diseases (INI/FIOCRUZ), a referral hospital in infectious diseases in Brazil, located in Rio de Janeiro. This hospital receives patients from all municipalities, mainly the metropolitan area. According to the last census conducted in 2010, Rio de Janeiro had a population of 6.320.446 inhabitants and the metropolitan region, which is composed of 21 municipalities and is the second largest metropolitan area in Brazil, had 11.812.482 inhabitants [24].

Stool samples were collected by the patient in plastic disposable flasks with and without preservatives and maintained at 4°C until laboratory analysis on the same day. Flasks were labeled with the name, collection date and the hospital number. The medical request came along with the sample. The parasitological tests were conducted at the Parasitology Laboratory of INI by experienced laboratory technologists. This laboratory is certified by the College of American Pathologists.

For laboratory diagnosis of *G*. *lamblia*, the fresh specimens were analyzed by means of centrifugation sedimentation [25] and centrifugal flotation in zinc sulphate solution [26]. Specimens preserved in MIF solution (mertiolate-iodine-formaldehyde) were processed by the centrifugation sedimentation method [25]. The slides were then observed under the microscope (Nikon Eclipse E200, magnification of 10 and 40X). In order to improve the detection of positive samples, a total of 262 randomly selected microscopy negative fecal samples were screened using the *Giardia lamblia* Antigen ELISA kit (Genway Biotech Inc., USA) according to the manufacturer’s instructions. All patients attending INI/FIOCRUZ are dewormed when diagnosed (drugs are provided by the institution itself).

### Molecular study

#### DNA extraction

The molecular analysis was performed only on samples without preservatives (n = 65). Approximately 5g of fecal sample, positive for *G*. *lamblia*, was washed with distilled water, filtered through doubled gauze and then centrifuged (1000 x *g* for two minutes). These procedures were repeated two times. The concentrated cysts were stored at -20°C until DNA extraction. Samples collected in 2011 and 2012 were subjected to DNA extraction in 2013, whereas samples collected from 2013 were extracted regularly within one month of collection. DNA extraction was performed using the QIAamp DNA Stool mini Kit (Qiagen, Germany) according to the manufacturer’s instructions. For PCR negative samples, a modified DNA extraction was implemented with minor modifications. In the first step, glass pearls and polyvinylpyrrolidone 10% solution was added and the incubation time was increased to one hour at 95°C; in the final steps, glycogen was added for DNA precipitation.

#### Nested-PCR

Extracted DNA was analyzed by nested-PCR using three *G*. *lamblia* gene loci: small-subunit ribosomal RNA (*ssu rRNA*) [27], β-giardin (*bg*) [28, 29] and glutamate dehydrogenase (*gdh*) [30]. Amplification of the *ssu rRNA* gene was performed with primers Gia2029 and Gia2150c in the primary PCR, and with RH11 and RH4 primers in the secondary reaction, generating a 292bp fragment [27] (Table 1). After an initial denaturation of 96°C for 4min, a set of 35 cycles was run, each consisting of 45s at 96°C, 30s of annealing (55°C for the primary reaction, 59°C for the second), and 45s at 72°C, followed by a final extension step of 4min at 72°C. The amplification of the *bg* gene was performed using a semi-nested PCR and a nested PCR protocols. The first amplification reaction was common to both PCR protocols generating a 753bp fragment using the primer pair G7 and G759 [28]. In the semi-nested PCR reaction, a fragment of 384bp was amplified using the primer pair G376 and G759 [28] and in the nested PCR a fragment of 511bp was amplified using the primer pair 2005F and 2005R [29] (Table 1). The primary and the semi-nested amplifications were carried out as follows: 1 cycle of 94°C for 5min, followed by 35 cycles of 94°C for 30s, 65°C for 30s and 72°C for 1min. A final extension of 72°C for 7min and a 4°C hold was used. The nested PCR was performed with the following amplification conditions: 1 cycle of 95°C for 15min, followed by 35 cycles of 95°C for 30s, 65°C for 30s and 72°C for 1min, and a final extension of 72°C for 7min. For the amplification of the 432pb fragment of *gdh* gene, a semi-nested PCR was done as described by Read *et al*. [30]. Primary PCR was run using the forward primer GDHeF and reverse primer GDHiR (Table 1). For secondary PCR, forward primer GDHiF and reverse primer GDHiR were used. The primary and the secondary reactions were performed under the following conditions: 1 cycle of 94°C for 2min, 56°C for 1min and 72°C for 2min, followed by 55 cycles of 94°C for 30s, 56°C for 20s and 72°C for 45s, and a final extension of 72°C for 7min.

**Table 1:** List and sequence of primers.

Locus	Primer	Sequence (5´-3´)	Reference
ssu rRNA*	Gia2029	AAGTGTGGTGCAGACGGACTC	[27]
	Gia2150c	CTGCTGCCGTCCTTGGATGT	
	RH11	CATCCGGTCGATCCTGCC	
	RH4	AGTCGAACCCTGATTCTCCGCCAGG	
bg*	G7	AAGCCCGACGACCTCACCCGCAGTGC	[28]
	G759	GAGGCCGCCCTGGATCTTCGAGACGAC	
	G376	CATAACGACGCCATCGCGGCTCTCAGGAA	
	2005F	GAACGAACGAGATCGAGGTCCG	[29]
	2005R	CTCGACGAGCTTCGTGTT	
gdh*	GDHeF	TCAACGTYAAYCGYGGYTTCCGT	[30]
	GDHiF	CAGTACAACTCYGCTCTCGG	
	GDHiR	GTTRTCCTTGCACATCTCC	
gdh**	gdhA_F	CCGGCAACGTTGCCCAGTTT	[8]
	gdhA_R	ACTTGTCCTTGAACTCGGA	
	gdhB_F	CGTATTGGCGTCGGCGGT	
	gdhB_R	TGTGGCCTCTGGTCTGATAG	
tpi**	tpiA_F	TCGTCATTGCCCCTTCCGCC	[8]
	tpiA_R	CGCTGCTATCCTCAACTG	
	tpiB_F	GATGAACGCAAGGCCAATAA	
	tpiB_R	GATTCTCCAATCTCCTTCTT	
*orf*C4**	orfC4A_F	CTGTAGACAGGGCCCAGGCC	[8]
	orfC4A_R	ATGATGTCCCCTGCCCTTAAT	
	orfC4B_F	ACTGTCCATTTCTATCTGAG	
	orfC4B_R	GGATTCCATTGGCCTCCACCT	

* Primers used in the nested-PCR;

** Primers used in the qPCR.

All reactions contained 12.5μL of NZYTaq 2x Green Master Mix (Nzytech, Portugal), 1μL of each primer (10pmol/μL), 1μL of extracted DNA and 8.5μL of sterile water, performing a final volume of 25μL. PCR was carried out on the MJ Mini™ Thermal Cycler (BioRad). In all PCR reactions, *Giardia*-positive DNA sample (strain WB, clone 6, ATCC 30957) and nuclease-free distilled water were used as positive and negative controls, respectively. The PCR products were analyzed on 1.5% agarose gels stained with ethidium bromide and visualized using a gel documentation system (Uvitec, UK).

#### qPCR

The extracted DNA was also analyzed by real-time quantitative assay (qPCR) using *gdh*, *tpi* and *orf*C4 (open reading frame C4) genes as previously described [8]. Amplification reactions contained 12.5μL of Sso Fast^™^ EvaGreen SuperMix (BioRad, USA), 1μL of each primer (10pmol/μL), 1μL of extracted DNA and 9.5μL of sterile water, for a final volume of 25μL. All reactions were performed in triplicate; positive and negative controls were also included in each PCR. Cycle threshold (Ct) values of >36 were considered to reflect limited reproducibility due to low copy numbers.

qPCR assays were performed on CFX 96^™^ Real Time PCR Detection System (BioRad) and the primers used are listed in Table 1. A minor modification was done in the thermal profile. All reactions started with a denaturation step at 98°C for 2min, followed by 40 cycles of denaturation (98°C for 5s) and annealing (59°C for 5s). The melting curve program was performed at the end of each reaction and consisted of 95°C for 5s, 65°C for 1min, and heating to 95°C with continuous acquisition (5 acquisitions per degree Celsius).

#### Genetic characterization of *G*. *lamblia*

The *bg* and *gdh* positive nested-PCR samples were subsequently analyzed by polymerase chain reaction-restriction fragment length polymorphisms (PCR-RFLP), in order to discriminate the *G*. *lamblia* genetic assemblages.

PCR-RFLP analysis was carried out by digesting the secondary PCR products of the nested-PCR of *bg* and *gdh* genes. The *bg* gene PCR products were digested with ten units of the endonucleases *Hha*I (384pb fragment) [28] and *Hae*III (511pb fragment) [29] (New England Biolabs Inc., USA) in a final volume of 20μL for 4h at 37°C. The amplified products of *gdh* gene were digested with two units of the endonucleases *Nla*IV or *Rsa*I (New England Biolabs Inc., USA) in a final volume of 20μL for 3h at 37°C [30]. Profiles were analyzed on 3% agarose gels stained with ethidium bromide and visualized using a gel documentation system (Uvitec, UK).

Additionally, *G*. *lamblia* A and B assemblage-specific primers targeting the *gdh*, *tpi* and *orf*C4 genes (Table 1) were used to genotype *G*. *lamblia* assemblages and to detect mixed infections of Assemblage A and Assemblage B, in qPCR assay [8].

### Statistical analysis

The data entry was carried out using Excel software and analyzed using Statistical Package for the Social Sciences (SPSS) version 16. Percentages were used to perform the exploratory analysis of the categorical variables and quantitative variables are presented as mean ± standard deviation (SD). Pearson´s chi-squared and Fisher’s Exact Test were used for categorical data. The level of statistical significance was set as p<0.05.

### Ethical statement

The Research Ethics Committee Evandro Chagas National Institute of Infectious Diseases (INI/FIOCRUZ) approved the use of the patients stool samples (protocol number 127.542). This project was in accordance with the Brazilian Ethical Resolutions, especially Resolution CNS 196/1996 and its complementary and the Code of Medical Ethics of 1988 (articles 122–1307). Written informed consent was obtained from all patients or legal guardians of patients younger than 18 years, prior to sample collection. The informed consent was provided after a detailed explanation of the objectives of the work. A term of privacy and confidentiality was signed by the researches for patients for whom it was not possible to obtain informed consent beforehand.

## Results

### Characteristics of study participants

During the study period, a total of 5179 patients were initially screened for the presence of intestinal parasites (helminths and protozoa) by direct microscopy. The detection of *G*. *lamblia* coproantigen was performed by ELISA on 262 patients out of 5081 microscopy negative samples. All *Giardia* positive samples (microscopically or ELISA) were confirmed by molecular methods. The prevalence of enteroparasites found in this study was described previously by our team (Faria *et al*., unpublished data). *G*. *lamblia* cysts were identified in 32 of the 1554 (2.1%) patients attending the hospital in 2011; 29 of 1374 (2.1%) patients in 2012; 17 of 1190 (1.4%) in 2013; 19 of 938 (2%) in 2014, and 1 of 123 (0.8%) in 2015. Overall, 98 patients were infected with *G*. *lamblia*, of whom 65 stool sample (without preservative) were included in further molecular analysis. The majority of the patients were adults (69.2%) with an average of 32.54±13.69 years of age (Mean±SD; median = 32). The highest *Giardia* prevalence occurred between 30–39 years (33.3%), and there were more male than female infected (69.2% *versus* 30.8%). Seventy-two percent of patients were educated above the primary; most of them (95.4%) live in metropolitan region and 61.5% live in Rio de Janeiro municipality.

### Coproantigen ELISA

Among the 262 *Giardia* negative samples on microscopy analysis only one (0.4%) was positive for *Giardia* coproantigen ELISA.

### Nested-PCR

Out of the 65 positive stool samples for *G*. *lamblia*, 13 (20%) were collected in 2011, 22 (33.8%) in 2012, 11 (16.9%) in 2013, 18 (27.7%) in 2014, and 1 (1.5%) in 2015 (Table 2). Among them, 60 (92.3%) were successfully amplified at the *ssu rRNA* gene, a multi copy gene, which largely confirms the microscopy results (Table 2). In relation to *bg* and *gdh*, single copy genes, 41 (63.1%) samples were successfully amplified by nested-PCR: 38 (58.5%) at the *bg* locus and 32 (49.2%) in *gdh* locus (Table 2).

**Table 2:** Summary of the results of nested-PCR and qPCR in *G*. *lamblia* positive samples.

Year	SamplesNo. (%)	Nested-PCR				qPCR				Amplified samples (nested-PCR + qPCR) No. (%)	Not amplified samples No. (%)
		*ssu rRNA*	*bg*	*gdh*	*bg*+*gdh*	*tpi*	*orf*C4	*gdh*	*gdh*+*tpi*+*orf*C4		
**2011**	13 (20)	11 (16.9)	6 (9.2)	2 (3.1)	7 (10.8)	7 (10.8)	6 (9.2)	3 (4.7)	7 (10.8)	11 (16.9)	2 (3.1)
**2012**	22 (33.8)	20 (30.8)	9 (13.8)	7 (10.8)	10 (15.4)	16 (24.6)	12 (18.5)	10 (15.4)	19 (29.2)	20 (30.8)	2 (3.1)
**2013**	11 (16.9)	11 (16.9)	9 (13.8)	8 (12.3)	9 (13.8)	10 (15.4)	9 (13.8)	9 (13.8)	10 (15.4)	11 (16.9)	0
**2014**	18 (27.7)	17 (26.2)	13 (20)	14 (21.5)	14 (21.5)	15 (23.1)	15 (23.1)	15 (23.1)	17 (26.2)	17 (26.2)	1 (1.5)
**2015**	1 (1.5)	1 (1.5)	1 (1.5)	1 (1.5)	1 (1.5)	0	1 (1.5)	0	1 (1.5)	1 (1.5)	0
**Total**	65 (100)	60 (92.3)	38 (58.5)	32 (49.2)	41 (63.1)	48 (73.8)	43 (66.2)	37 (56.9)	54 (83.1)	60 (92.3)	5 (7.7)

*ssu rRNA*, multi copy gene; *bg*, *gdh*, *tpi* and *orfC4*, single copy genes.

We could observe that nested-PCR efficiency was dependent on the sampling years. Amplification were less effective in samples collected in 2011 and 2012, only 31 samples of 35 (88.6%) were PCR-positive, whereas 29 samples of 30 (96.7%) were PCR-positive in subsequent years (Table 2).

Twenty-nine samples (44.6%) were amplified in three loci, 12 were amplified in two loci (18.5%) and 19 (29.2%) were only amplified in one locus (S1 Table).

### qPCR

After the analysis by qPCR assay, a total of 54 (83.1%) samples were amplified using at least one locus (Table 2). This technique was more sensitive than nested-PCR of *bg* and *gdh* detecting more 13 samples (20%). All positive samples in qPCR showed a Ct value less than 35 (ranged from 24 to 35) (S2 Table). The *tpi* product was amplified in 48 samples (73.8%), followed by the *orf*C4 gene in 43 (66.2%) and the *gdh* gene in 37 samples (56.9%) (Table 2). As with nested-PCR, qPCR efficiency of the samples varied according to the collection year and time interval between collection and DNA extraction. Samples collected in 2011 and 2012 showed a lower efficiency (26 of 35, 74.3%) compared with samples from 2013 (28 of 30, 93.3%) (Table 2).

The three loci were amplified in 33 samples (50.8%), two loci in 8 (12.3%) samples and one locus in 13 samples (20%). The qPCR was negative in eleven samples (S1 Table).

### Nested-PCR and qPCR

Nested-PCR and qPCR techniques successfully amplified 60 (92.3%) samples using at least one locus (Table 2). The amplification rates of the five markers used were 92.3%, 58.5%, 64.6%, 73.8%, 66.2% for the *ssu rRNA*, *bg*, *gdh*, *tpi* and *orf*C4 genes, respectively. The *ssu rRNA* gene, a multi copy gene, showed a better amplification rate by nested-PCR. In relation to the single copy genes, *tpi* showed better results by qPCR (Table 2). Regarding *gdh* gene, we could detect that 32 (49.2%) samples were successfully amplified in conventional PCR, 37 (56.9%) in qPCR, totaling 42 (64.6%) amplified samples.

All five loci were amplified in 26 (40%) samples, four loci in 15 (23.1%), three loci in 10 (15.4%), two loci in 4 (6.2%), and a locus gene in 5 (7.7%). Six samples (9.2%) were only amplified using nested-PCR (INI 6, 29, 37, 38, 42, and 46), 54 (83.1%) were amplified by both techniques (PCR + qPCR) and 5 (7.7%) samples were negative in all five loci despite repeated trials (INI 5, 22, 36, 40, 63) (S1 Table). Most of the negative samples were collected between the years 2011 (2 samples) and 2012 (2 samples) (Table 2).

### Genotyping analysis of *G*. *lamblia*

#### PCR-RFLP

The *G*. *lamblia* assemblage was successfully determined on 41 samples by PCR-RFLP at the *bg* and *gdh* locus gene: Assemblage A was found in 21 samples (51.2%) and Assemblage B in 20 samples (48.8%) (Table 3). Among the 38 samples amplified by *bg* gene, Assemblage A was identified in 21 (55.3%) patients and Assemblage B in 17 (44.7%). At the *gdh* locus, Assemblage A was observed in 13 (40.6%) samples and Assemblage B in 19 (59.4%) (Table 3).

**Table 3:** Genotypic characterization of *G*. *lamblia* by PCR-RFLP and Assemblage A- and B-specific qPCR.

Assemblage	PCR-RFLP			qPCR				Genotyped samples (PCR-RFLP + qPCR) No. (%)
	*bg*	*gdh*	Total (*bg*+*gdh*)	*gdh*	*tpi*	*orf*C4	Total (*gdh*+*tpi*+*orf*C4)	
**A**	21 (55.3)	13 (40.6)	21 (51.2)	20 (54.1)	27 (56.3)	22 (51.2)	28 (51.9)	29 (52.7)
**B**	17 (44.7)	19 (59.4)	20 (48.8)	17 (45.9)	21 (43.7)	21 (43.7)	26 (48.1)	26 (47.3)
**Total**	38	32	41	37	48	43	54	55

Restriction patterns showed that 16 samples are sub-assemblage AII, and no sub-assemblage AI was identified; 7 were classified as BIII, 4 as BIV and 8 mixed infection (BIII + BIV); no mixed infection with the two Assemblages A and B was identified (Table 4).

**Table 4:** Genotypic characterization of *G*. *lamblia* sub-assemblages by PCR-RFLP.

Sub-assemblage	PCR-RFLP		
	*bg*	*gdh*	Total (*bg*+*gdh*)
**AII**	16	13	16
**BIII**	-	7	7
**BIV**	-	4	4
**BIII + BIV**	-	8	8

#### qPCR assemblage-specific

The qPCR was used to allow a more accurate detection of mixed infections by the two Assemblages (A + B) in clinical samples. At the *gdh* locus, Assemblage A was observed in 20 (54.1%) patients and Assemblage B in 17 (45.9%). At the *tpi* gene, 27 (56.3%) samples belonged to Assemblage A and 21 (43.7%) belonged to Assemblage B. Similar results were observed at *orf*C4 locus, where Assemblage A was identified in 22 (51.2%) samples and Assemblage B in 21 (48.8%). Regarding the three markers (*gdh*, *tpi*, *orfC4*), 28 (51.9%) samples belonged to Assemblage A, 26 (48.1%) to Assemblage B and no mixed infection (A + B) was detected (Table 3).

#### PCR-RFLP and qPCR assemblage-specific

The PCR-RFLP and qPCR results showed 100% concordance in typing *G*. *lamblia* isolates (Table 3). Although more sensitive, qPCR was unable to subtyping samples. Twenty-nine Assemblage A and twenty-six Assemblage B samples were identified by PCR-RFLP and qPCR, totalizing 55 (84.6%) genotyped samples (Table 3). At the *gdh* locus, 42 samples were amplified using the nested-PCR and qPCR: Assemblage A was observed in 20 patients and Assemblage B in 22, being the gene that detected a larger number of samples belonging to Assemblage B (S1 Table).

Due to amplification difficulties, not all samples were genotyped at the sub-assemblages level; 5 samples (INI 14, 15, 30, 32, 37) belonged to Assemblage A and one (INI 34) to Assemblage B (S1 Table).

Analyzing the assemblages prevalences over the four years of this study, it is possible to observe in 2011 and 2012 the Assemblage A was predominant (S1 Table). In 2011 six samples belonged to Assemblage A and two belonged to Assemblage B, and in 2012, 14 samples were Assemblage A and 5 were Assemblage B. However, in subsequent years there was a predominance of Assemblage B: in 2013 three samples belonged to Assemblage A and seven belonged to Assemblage B; in 2014 six samples were Assemblage A and 11 were Assemblage B; in 2015 the sample belongs to Assemblage B.

#### Socio-demographic profile of genetic assemblages

There were no statistical differences between Assemblages A and B regarding residence, gender and age distribution (p>0.05) (Table 5). However, Assemblage B had a slight prevalence between 30–39 year of age; probably due to a larger number of patients in this age group. As expected, Rio de Janeiro municipality had a greater number of patients (40 samples; 61.5%) infected with *G*. *lamblia* due to its larger population.

**Table 5:** Distribution of *G*. *lamblia* assemblages according to age groups, sex, housing municipality and sampling year.

Characteristic	Assemblage A (n = 29)	Assemblage B (n = 26)	Total
	No. (%)%	No. (%)	
***Age (years old)***			
0–9	1 (3.5)	4 (15.4)	5
10–19	3 (10.3)	1 (3.8)	4
20–29	8 (27.6)	5 (19.2)	15
30–39	7 (24.1)	12 (46.2)	21
40–49	6 (20.7)	3 (11.5)	12
50–59	3 (10.3)	1 (3.8)	6
Missing	1 (3.5)	-	2
***Gender***			
Female	9 (31)	7 (26.9)	20
Male	20 (69)	19 (73.1)	45
***Municipality***			
Belford Roxo	1 (3.5)	-	1
Caxias	4 (13.8)	4 (15.4)	9
Nilópolis	-	1 (3.8)	1
Nova Iguaçu	3 (10.3)	5 (19.2)	9
Rio de Janeiro	18 (62.1)	15 (57.7)	40
Santo Antônio de Pádua	-	1 (3.8)	1
São Gonçalo	1 (3.5)	-	1
São João de Meriti	1 (3.5)	-	1
Missing	1 (3.5)	-	2
***Year of collection***			
2011	6 (20.7)	2 (7.7)	13
2012	14 (48.3)	5 (19.2)	22
2013	3 (10.3)	7 (26.9)	11
2014	6 (20.7)	11 (42.4)	18
2015	-	1 (3.8)	1

## Discussion

Despite the high prevalence of giardiasis in Brazil, there is a lack of information on the genetic diversity of *G*. *lamblia*. So far only one study was performed with human samples in Rio de Janeiro and the genetic data was based just on the *bg* locus gene. The purpose of this study was to determine the prevalence of *G*. *lamblia* assemblages in patients attending the Evandro Chagas National Institute of Infectious Diseases (FIOCRUZ, Rio de Janeiro), using four different molecular markers.

Our study detected the presence of Assemblages A (52.7%) and B (47.3%), with a slight predominance of the first. It is the first time that Assemblage B was observed in human clinical samples in Rio de Janeiro. The previous study performed in Rio de Janeiro with human isolates reported only the presence of Assemblage A [23]. The distribution of Assemblage A and Assemblage B varies greatly from one country to another and sometimes within the same country. Surely, due to the enormous expanse of territory of Brazil, prevalence of the assemblages varies between regions and contrasting data are observed. The majority of *G*. *lamblia* infections identified in Fortaleza [15], Minas Gerais [16], Paraná [18] and São Paulo [19] were Assemblage B. Conversely, a study from São Paulo observed that Assemblage A was more prevalent [14]. Recently, David *et al*. [12] and Durigan *et al*. [17] showed no significant difference between assemblages distribution, being similar to findings by our group. In Latin America, Ramírez *et al*. [31] reported that Assemblage B was predominant in Colombia, while in México Torres-Romero *et al*. [32] note predominance of Assemblage A and in Cuba Puebla *et al*. [33] found that Assemblages A and B were detected at equal frequencies.

In our study, a change in the genetic profile over the years could be observed as well. Assemblage A was most prevalent until the year 2012, while in the subsequent years (2013–2015) there was an increased number of cases of Assemblage B. Kohli *et al*. [15] reported that children with Assemblage B shed significantly more cysts than children infected with Assemblage A. This coupled with fecal-oral transmission, may contribute to the higher prevalence and dispersion of Assemblage B. We observed a predominance of Assemblage B in adults between the ages 30 to 39 years, although this may be due to a greater number of patients in this age group. A study conducted in England [34] also detected a higher prevalence of Assemblage B in adults in their 30s and 40s and a predominance of Assemblage A in older people (≥70 years old. Though we cannot draw any conclusions with the results obtained here, further research is needed in order to evaluate the possible aforementioned association.

Regarding Assemblage A, the samples that were genotyped at the sub-assemblage level (16 of 29; 55.2%) were characterized as sub-assemblage AII, an anthroponotic genotype [6]. Similarly, previous works about human giardiasis in Brazil showed that sub-assemblage AII was identified more often than sub-assemblage AI [12, 14, 17–19]. Nevertheless, these results contrast once more with the data obtained in Rio de Janeiro [23], where 97% of the samples were identified as sub-assemblage AI, a genotype commonly found in animals. Besides changes in frequency of infections with different assemblages over time, we could also observe a switch-over in the sub-assemblages, where sub-assemblage AI was not detected and sub-assemblage AII became more frequent. Among Assemblage B, our study observed a greater number of patients with sub-assemblage BIII, which is not in agreement with previous results from Brazil, where sub-assemblage BIV was identified more often [17–19]. The detection of sub-assemblages AII, BIII and BIV in the clinical samples of patients from Rio de Janeiro suggest that transmission of giardiasis may be mainly person-to-person (directly or indirectly by water or food), since both assemblages are predominant in humans. This hypothesis is also supported by the absence of sub-assemblage AI, mostly found infecting companion animals and livestock [5], in patients of our study.

The prevalence of each assemblage varies from region to region, but Assemblage B seems to be more common in Brazil and in other countries than Assemblage A [1, 5, 6]. *G*. *lamblia* assemblages are not evenly distributed, and the reasons are still unclear. Possibly, the distribution is not geographically associated and may be explained by parasite factors (such as the rate of multiplication, variable surface proteins expressed, resistance to pharmaceuticals, and ability to invade immune response), host factors (such as immune status, history of exposure, diet, and concomitant intestinal microbiota), different transmission routes and infection sources [1, 35]. In addition, the preferential amplification of one particular locus gene could also account for the differences in prevalence of assemblages [5, 8].

Even though no significant statistical difference was observed between assemblages distribution (52.7% Assemblage A and 47.3% Assemblage B), mixed infections with both Assemblages (A + B) was not detected. However, intra-assemblage mixed infections were observed in Assemblage B (BIII+BIV). The presence of more than one assemblage infecting one patient has been previously recorded in Brazil [15, 17] and in other countries [1]. Sometimes the failure to detect these mixtures is not due to the absence of the mixed infection, but because one assemblage can be preferentially amplified over another at one locus [6]. To prevent such thing we used qPCR assemblage-specific [8], and confirmed that mixed infection were not present in the studied samples. The qPCR technique also improved the *G*. *lamblia* detection, in relation to nested-PCR of *bg* and *gdh* genes. The increased specificity and sensitivity, as well as simultaneous detection of Assemblages A and B are benefits that qPCR provided in the present study.

Additionally, genotyping by PCR-RFLP and qPCR were identical and no divergence or inconsistencies in the assemblages were found among the four different loci. It is important to highlight that none out of 49 samples, simultaneously positive at two, three or four loci, had incongruent genotyping results (each other). So we are very confident that the assemblage’s profile of the samples successfully characterized represents the genetic population of *G*. *lamblia* in Rio de Janeiro. These results are in contrast with previous reports where incongruent genotyping results have been reported [5, 36, 37]. However, the use of several molecular markers (MLG) allowed us to considerably increase the detection of *G*. *lamblia* assemblages and to determine, in a reliable manner, the distribution of assemblages across the population.

The *ssu rRNA* gene is strongly conserved and due their multicopy nature, the PCR that targets this locus has a high sensitivity [9, 38]. Thus, to ensure the presence or absence of *G*. *lamblia* DNA, the *ssu rRNA* gene was used. Among the 65 positive samples, we could detect *G*. *lamblia* DNA in 60 samples, largely confirming the microscopy results.

Nevertheless, our molecular approach did not amplify five samples (7.7%) (false-negatives). Therefore we concluded that DNA degradation, insufficient DNA or inhibition of PCR could have limited the success of the amplification. The stool samples were stored without preservatives at -20°C before DNA extraction. Probably the freezing for several months (or years) may have led to degradation of the cysts/DNA of *G*. *lamblia*. Minetti *et al*. [34] had similar results, where the overall PCR amplification success of the samples varied accordingly to the year of collection and the time from collection to DNA extraction. Specimens collected recently (extracted within one month) showed a higher amplification success rate compared with amplification of older specimens. Many researchers have reported the difficulty of typing assemblages, even in the microscopically positive samples. Typically, the failure in amplifying is related to the nature of the biological sample used, DNA ineffective extraction and DNA degradation. Stool samples contain PCR inhibitors, such as bilirubin, bile salts, hemoglobin, phenolic compounds, and complex polysaccharides, which are co-purified together during DNA extraction. It also has enormous quantity of non-specific DNA and the potential for low numbers of cysts. Prolonged storage of the stool samples also take into consideration, old samples may possible have degradation of genetic material [39, 40].

Moreover, the DNA region between the PCR primers and the genomic sequences could be degraded or there was nucleotide mismatches, that may cause a strong reduction or even a lack of amplification, specially taking into consideration that *bg*, *gdh*, *tpi* and *orf*C4 genes were single copy [41]. In negative samples, new DNA extraction was performed with minor modifications: addition of glass beads and a solution of polyvinylpyrrolidone (PVP) in the first step; increasing the incubation time (5 minutes for 1 hour at 95°C); and addition of glycogen for DNA precipitation. Despite this procedure the samples remained negative.

The detection of *G*. *lamblia* by microscopy has been demonstrated to be less sensitive compared to ELISA [1]. According to Sommer *et al*. [42], *Giardia* cysts are eliminated along with the feces intermittently, which make the coproantigen ELISA the most reliable method for detection of an infection with this protozoan parasite. Conversely, a recent study comparing rapid diagnostics methods observed ELISA false-positive for *G*. *lamblia* [43]. In our study, among the 262 *Giardia* negative samples analyzed by microscopy, only one (0.4%) was positive for *Giardia* coproantigen ELISA and was not amplified by molecular methods (false-positive). Perhaps the patient's immune system eliminated the parasite, having only the release of antigens in feces. These results allowed us to conclude that collecting three fecal specimens on alternate days, the use of cysts concentration technique and microscopy observation by experienced laboratory technologists is an appropriate approach for *G*. *lamblia* detection.

This is the first description of genetic characterization of *G*. *lamblia* in Brazil using *bg*, *gdh*, *tpi* and *orf*C4 genes, and it is also the first time that Assemblage B of *G*. *lamblia* was reported in human clinical samples from Rio de Janeiro. A switch in genetic profile over the years was observed, firstly predominance of Assemblage A and lastly of Assemblage B. The frequency of sub-assemblages has changed completely, where AI disappeared and mixed infection within Assemblage B was detected. Obviously, the detection of anthroponotic sub-assemblages (AII, BIII and BIV) does not exclude the investigation of the role of animals in the dynamic of transmission, especially because information on *G*. *lamblia* isolated from animals of Rio de Janeiro (and others States in Brazil) are scarce. Few studies have been performed in Brazil regarding assemblages of *G*. *lamblia* in samples obtained from humans and animals. Further studies using accurate molecular typing are imperative for unraveling the intricate epidemiology of giardiasis.

## Supporting Information

S1 Table Genotyping of 65 *G*. *lamblia* positive samples based on *ssu rRNA*, *bg*, *gdh*, *tpi* and *orf*C4 genes. (DOCX)

S2 Table Threshold cycle values. (DOCX)
