# Supplementary material for: Molecular Characterization of Giardia lamblia: First Report of Assemblage B in Human Isolates from Rio de Janeiro (Brazil)
Source: PLoS One. 2016 Aug 12;11(8):e0160762. doi: 10.1371/journal.pone.0160762 (PMC4982690; doi:10.1371/journal.pone.0160762)
Supplement: S1 Table — (DOCX) [file pone.0160762.s001.docx]

**S1 Table. Genotyping of 65 *G. lamblia* positive samples based on *ssu rRNA, bg*, *gdh*, *tpi* and *orf*C4 genes.**

| Year | Isolates (ID) | Genes | | | | | |
| --- | --- | --- | --- | --- | --- | --- | --- |
|  |  | ***ssu rRNA*** | ***bg*** | ***gdh*** | | ***tpi*** | ***orf*C4** |
|  |  | **Nested-PCR** | **PCR-RFLP** | **PCR-RFLP** | **qPCR** | **qPCR** | **qPCR** |
| 2011 | INI 30 | **Pos** | **A** | Neg | **A** | **A** | **A** |
|  | INI 31 | **Pos** | Neg | Neg | Neg | **B** | **B** |
|  | INI 32 | **Pos** | **A** | Neg | Neg | **A** | **A** |
|  | INI 33 | **Pos** | Neg | **BIII** | **B** | **B** | **B** |
|  | INI 35 | **Pos** | **AII** | Neg | Neg | **A** | Neg |
|  | INI 37 | **Pos** | **A** | Neg | Neg | Neg | Neg |
|  | INI 39 | **Pos** | **AII** | Neg | Neg | **A** | **A** |
|  | INI 41 | **Pos** | **AII** | **AII** | **A** | **A** | **A** |
|  | INI 29, 38, 42 | **Pos** | Neg | Neg | Neg | Neg | Neg |
|  | INI 36, 40 | Neg | Neg | Neg | Neg | Neg | Neg |
| 2012 | INI 1, 7, 8, 12 | **Pos** | Neg | Neg | **A** | **A** | **A** |
|  | INI 2, 3 | **Pos** | Neg | Neg | Neg | **A** | Neg |
|  | INI 4 | **Pos** | Neg | Neg | **A** | Neg | Neg |
|  | INI 10 | **Pos** | **B** | **BIII + BIV** | **B** | **B** | **B** |
|  | INI 11, 17, 19, 23 | **Pos** | **AII** | **AII** | **A** | **A** | **A** |
|  | INI 13 | **Pos** | Neg | **BIII** | Neg | Neg | **B** |
|  | INI 14, 15 | **Pos** | **A** | Neg | Neg | **A** | Neg |
|  | INI 21 | **Pos** | **B** | **BIV** | Neg | Neg | **B** |
|  | INI 24 | **Pos** | Neg | Neg | Neg | **B** | Neg |
|  | INI 25 | **Pos** | Neg | Neg | Neg | **A** | **A** |
|  | INI 34 | **Pos** | **B** | Neg | Neg | **B** | Neg |
|  | INI 5, 22 | Neg | Neg | Neg | Neg | Neg | Neg |
|  | INI 6 | **Pos** | Neg | Neg | Neg | Neg | Neg |
| 2013 | INI 26 | **Pos** | Neg | Neg | **B** | **B** | **B** |
|  | INI 27 | **Pos** | **B** | **BIV** | **B** | **B** | **B** |
|  | INI 28, 43 | **Pos** | **B** | **BIII** | **B** | **B** | **B** |
|  | INI 44 | **Pos** | **AII** | Neg | **A** | **A** | **A** |
|  | INI 45, 47 | **Pos** | **AII** | **AII** | **A** | **A** | **A** |
|  | INI 48 | **Pos** | **B** | **BIV** | **B** | **B** | Neg |
|  | INI 49 | **Pos** | **B** | **BIII** | Neg | **B** | **B** |
|  | INI 50 | **Pos** | **B** | **BIII + BIV** | **B** | **B** | **B** |
|  | INI 46 | **Pos** | Neg | Neg | Neg | Neg | Neg |
| 2014 | INI 51, 52, 58, 59, 60, 66 | **Pos** | **AII** | **AII** | **A** | **A** | **A** |
|  | INI 53, 57 | **Pos** | **B** | **BIII** | **B** | **B** | **B** |
|  | INI 54, 61, 67 | **Pos** | **B** | **BIII + BIV** | **B** | **B** | **B** |
|  | INI 55, 62 | **Pos** | Neg | Neg | **B** | **B** | **B** |
|  | INI 56 | **Pos** | Neg | Neg | Neg | Neg | **B** |
|  | INI 64 | **Pos** | **B** | **BIII + BIV** | Neg | **B** | Neg |
|  | INI 65 | **Pos** | Neg | **BIV** | **B** | **B** | Neg |
|  | INI 68 | **Pos** | **B** | **BIII + BIV** | **B** | Neg | **B** |
|  | INI 63 | Neg | Neg | Neg | Neg | Neg | Neg |
| 2015 | INI 69 | **Pos** | **B** | **BIII + BIV** | Neg | Neg | **B** |

*Pos*, positive

*Neg*, negative
