# Supplementary material for: Molecular Characterization of Giardia lamblia: First Report of Assemblage B in Human Isolates from Rio de Janeiro (Brazil)
Source: PLoS One. 2016 Aug 12;11(8):e0160762. doi: 10.1371/journal.pone.0160762 (PMC4982690; doi:10.1371/journal.pone.0160762)
Supplement: S2 Table — (DOCX) [file pone.0160762.s002.docx]

**S2 Table. Threshold cycle values.**

| **Isolates (ID)** | **Genes** | | |
| --- | --- | --- | --- |
|  | ***gdh*** | ***tpi*** | ***orfC4*** |
| INI 1 | 34 | 31 | 30 |
| INI 2 |  | 33 |  |
| INI 3 |  | 31 |  |
| INI 4 | 29 |  |  |
| INI 7 | 33 | 33 | 31 |
| INI 8 | 33 | 32 | 33 |
| INI 10 | 32 | 32 | 30 |
| INI 11 | 32 | 30 | 31 |
| INI 12 | 33 | 32 | 33 |
| INI 13 |  |  | 34 |
| INI 14 |  | 33 |  |
| INI 15 |  | 33 |  |
| INI 17 | 27 | 26 | 27 |
| INI 19 | 26 | 28 | 26 |
| INI 21 |  |  | 32 |
| INI 23 | 31 | 30 | 30 |
| INI 24 |  | 32 |  |
| INI 25 |  | 32 | 34 |
| INI 26 | 32 | 32 | 34 |
| INI 27 | 30 | 30 | 27 |
| INI 28 | 32 | 31 | 30 |
| INI 30 | 33 | 31 | 31 |
| INI 31 |  | 33 | 32 |
| INI 32 |  | 33 | 34 |
| INI 33 | 31 | 33 | 33 |
| INI 34 |  | 35 |  |
| INI 35 |  | 31 |  |
| INI 39 |  | 29 | 30 |
| INI 41 | 31 | 30 | 30 |
| INI 43 | 32 | 28 | 27 |
| INI 44 | 29 | 28 | 29 |
| INI 45 | 30 | 29 | 30 |
| INI 47 | 32 | 30 | 30 |
| INI 48 | 33 | 33 |  |
| INI 49 |  | 34 | 34 |
| INI 50 | 34 | 35 | 32 |
| INI 51 | 27 | 24 | 26 |
| INI 52 | 26 | 24 | 25 |
| INI 53 | 32 | 31 | 28 |
| INI 54 | 33 | 33 | 33 |
| INI 55 | 34 | 35 | 34 |
| INI 56 |  |  | 35 |
| INI 57 | 32 | 32 | 30 |
| INI 58 | 31 | 27 | 31 |
| INI 59 | 30 | 28 | 29 |
| INI 60 | 30 | 28 | 32 |
| INI 61 | 29 | 28 | 27 |
| INI 62 | 31 | 32 | 31 |
| INI 64 |  | 35 |  |
| INI 65 | 33 | 33 |  |
| INI 66 | 30 | 28 | 29 |
| INI 67 | 32 | 35 | 33 |
| INI 68 | 32 |  | 33 |
| INI 69 |  |  | 32 |
